# Vacuolar-type proton ATPase is required for maintenance of apicobasal polarity of embryonic visceral endoderm

**DOI:** 10.1038/s41598-021-98952-3

**Published:** 2021-09-29

**Authors:** Ge-Hong Sun-Wada, Hiroyuki Tabata, Yoh Wada

**Affiliations:** 1 Department of Biochemistry, Faculty of Pharmaceutical Sciences, Doshisha Women’s College of Liberal Arts, Kohdo, Kyotanabe, Kyoto 610-0395 Japan; 2 Division of Biochemistry, Faculty of Pharmaceutical Sciences, Himeji Dokkyo University, 7-2-1 Kami-ohno, Himeji, Hyogo 670-8524 Japan; 3 Division of Biological Sciences, Institute of Scientific and Industrial Research, Osaka University, Mihogaoka 8-1, Ibaraki, Osaka 567-0047 Japan

**Keywords:** Cell polarity, Endosomes, Embryology, Gastrulation

## Abstract

The endocytic compartments keep their interior acidic through the inward flow of protons and anions from the cytosol. Acidification is mediated by a proton pump known as vacuolar-type ATPase (V-ATPase) and transporters conferring anion conductance to the organellar membrane. In this study, we analysed the phenotype of mouse embryos lacking the V-ATPase c-subunit. The mutant embryos differentiated embryonic epithelial tissues, primitive endoderm, epiblast, and extraembryonic ectoderm; however, the organisation of these epithelia was severely affected. The apical-basal polarity in the visceral endoderm layer was not properly established in the mutant embryos, resulting in abnormal epithelial morphology. Thus, the function of V-ATPase is imperative for the establishment and/or maintenance of epithelial cell polarity, which is required for early embryogenesis.

## Introduction

Prior to the initiation of gastrulation, the mouse embryo is a cup-shaped structure comprising the visceral endoderm (VE) and epiblast. The VE is involved in nutrient uptake and transport, and plays an essential role in epiblast patterning that gives rise to the foetus. Both nutritional and patterning functions largely rely on the endocytic pathway of VE cells^1–4^.

The VE exhibits clear polarity; its apical surface faces the maternal circulation, whereas the basal side faces the embryo proper. The apical-basal cell polarity is established by sets of evolutionarily conserved polarity proteins, including atypical protein kinase C and Par proteins (aPKC-Par system)^5^. In addition to the aPKC-Par “master regulator,” membrane trafficking pathways are also important for the apical-basal polarisation program. The polarised setting of specific machineries to the apical or basal cell membrane demands development of distinctive transport mechanisms for newly synthesised molecules toward distinctive surface domains^6^. In addition to secretory trafficking, the endocytic pathway is implicated in the establishment and maintenance of cell polarity^7^. Internalisation of specific membrane domains into the cytoplasm and selective relocation of the membrane and soluble contents back to the cell surface are common for all cell types.

The endocytic and secretory compartments keep their interior acidic by an inward flow of protons and anions from the cytosol. Acidification is mediated by an active proton pump known as vacuolar-type ATPase (V-ATPase) and an array of transporters conferring anion conductance to the organellar membrane^8,9^. V-ATPase is a multi-subunit enzyme that uses energy from ATP hydrolysis to transport protons across membranes. It consists of two major functional domains, namely, V_1_ and V_0_. The former has eight different subunits (A, B, C, D, E, F, G, and H) and contains three catalytic sites for ATP hydrolysis formed by the A and B subunits^10–12^. The membrane-bound V_0_ domain is responsible for proton translocation across membranes. The V_0_ domain contains up to six subunits, including a, d, and e, and the proteolipids c, c’, and c”^10–12^, as well as accessory proteins ap1/Ac45 and ap2/(pro)renin receptor^13,14^. Proteolipids are small, four-pass transmembrane proteins that have both termini in the organelle lumen. Most of the subunits are encoded by multiple genetic loci; in contrast, the c-subunit and ap2 are encoded by a single locus^14,15^. Therefore, the genetic deletion of *Atp6v0c*, the locus encoding the c-subunit, results in the inactivation of all V-ATPase functions in various subcellular compartments, including endosomes and the Golgi, as well as the plasma membrane^16,17^.

De-acidification of the endocytic compartments leads to severe dysfunction in membrane trafficking in the secretory and endocytic pathways^18,19^. In the early endosome, recruitment of a small GTP-binding protein Arf6 and its guanine-nucleotide exchange factor ARNO necessitates the interaction with V-ATPase itself. This molecular interaction is dependent on the acidification inside the endosomes^20,21^. At the later stage of the endocytic pathway, the interaction between V-ATPase and RAB7 small GTP-binding protein was shown to be responsible for the cytoplasmic localisation of secretory lysosomes^22^.

Evidence suggests that V-ATPase is also involved in epithelial integrity^23–25^, indicating the tight coupling of acidification and cell polarity. In this study, we found that the apical-basal polarity of the VE epithelium was not properly maintained in *Atp6v0c* mutant embryos, resulting in abnormal tissue morphology. These observations indicate that V-ATPase function is imperative for the establishment and/or maintenance of epithelial cell polarity during early embryogenesis.

## Results

### Generation of *Atp6v0c* null mutant

In our previous study, we found that the genetic ablation of the V-ATPase c-subunit results in the loss of the embryo before gastrulation^16^. Mutant embryos lacking proteolipid c are implanted in the uterine epithelium but die shortly after^16^. An eventful developmental program proceeds during this stage of 4–6 days after fertilisation (E4.0–6.0)^26^. Mouse embryos acquire the basic architecture, including an anterior–posterior body axis and extraembryonic and embryonic structures. We have shown that endocytosis plays essential regulatory roles^2–4,27^. We investigated whether V-ATPase, which is directly committed to the endolysosomal system of various cell types, participates in this developmental program. Several previous studies on early embryo development have revealed mechanistic and molecular details of early embryogenesis^28^, enabling us to extend the findings of a previous study^16^ and examine the details of developmental defects associated with the loss of the c-subunit of V-ATPase.

We created an allele of the *Atp6v0c* locus where the lox P elements were placed at the intron 1–2 and 3′-untranslated region. We introduced it into the mouse genome by the ES cell-mediated homologous recombination to generate mice with *Atp6v0c*^*targeted*^ (Supplementary Fig. S1). As exons 2 and 3 encode 62 and 67 of the total 155 amino acid residues, respectively, their deletion is most likely to result in a loss of function of the gene product; therefore, we consider the resultant allele to be null, and termed it as *Atp6v0c*^*–*^. By crossing the *Atp6v0c*^*targeted/*+^ mice to mice transgenic for the EIIa-Cre recombinase expression gene^29^, we generated a mouse strain with the *Atp6v0c*^–^ allele.

### *Atp6v0c* deficiency resulted in the loss of the apical-basolateral organisation in the VE around E5.5

*Atp6v0c* deletion caused embryonic lethality; we were unable to obtain live pups homozygous for the null allele, whereas wild-type and heterozygous progenies were obtained at a ratio of 1:2 (Table 1). The mutant embryos could not survive beyond E5.0 ~ E5.5 (Table 1), i.e., soon after implantation. This result is in line with our previous observations on a different null allele of *Atp6v0c*^15,16^.

**Table 1:** Genotypes of progeny from *Atp6v0c*^+*/−*^ mating.

Age	*Atp6v0c* genotype				
	+/+	+/*−*	*−*/*−*	Resorptions	Chi-Square Test
E 3.5	9 (20%)	23 (52%)	12 (27%)	0 (0%)	*p* = 0.78
E 4.5	16 (26%)	30 (49%)	15 (25%)	0 (0%)	*p* = 0.98
E 5.0	21 (28%)	40 (53%)	7 (9%)	7 (9%)	*p* = 0.02
E 5.5	109 (25%)	234 (54%)	70 (16%)	40 (9%)	*p* = 0.0006
3–4 wks	47 (29%)	114 (71%)	0 (0%)	0 (0%)	*p* = 9.7 × 10^–13^

Timed mating was set up, and the isolated embryos or tails were used to obtain the genomic DNA. We were unable to genotype the resorptions.

**Figure 1:**
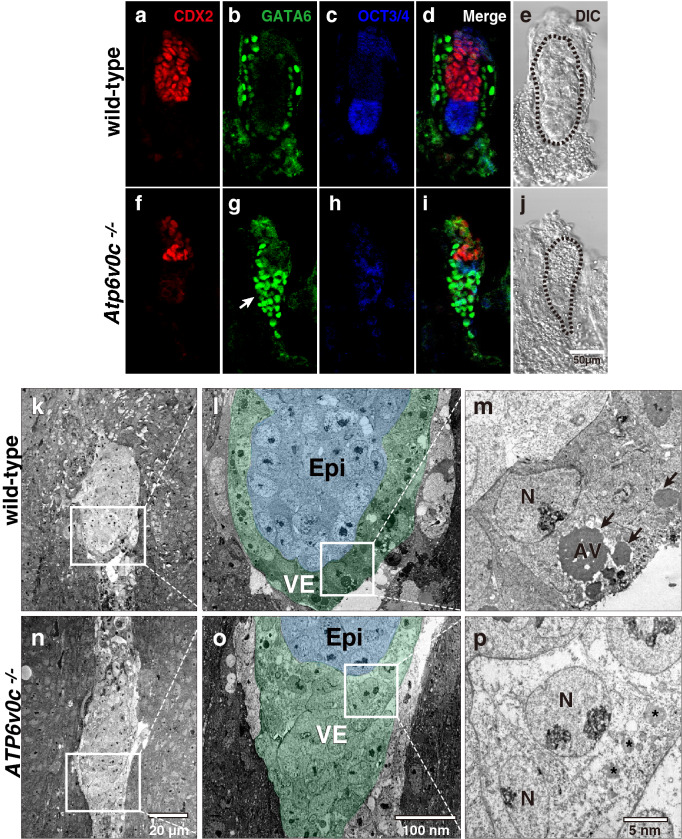
Immunofluorescence analysis of E5.2 embryos with various differentiation markers. The extraembryonic ectoderm (ExE) marker CDX2 (red, **a** and **f**), endoderm marker GATA6 (green, **b** and **g**), and epiblast (Epi) marker OCT3/4 (blue, **c** and **h**) are used. The merged (**d** and **i**) and DIC images (e and j) are also shown. The endoderm ExE and epiblast differentiation was observed in *Atp6v0c* mutant embryos, but their size reduced (**f** to **j**). The layer of GATA6-positive cells often appeared as a clump (white arrow). The outlines of embryos are indicated with a broken line. Representative images of wild-type (n = 18) and mutant (n = 3) embryos were shown. Scale bar, 50 µm. Transmission electron microscopy of E5.5 decidua (**k** to **p**). The pseudocoloured images of the visceral endoderm (VE) and Epi are shown (**l** and **o**). Apical vacuoles (AVs) are shown by black arrows (**m**). N; nucleus. The fragmented vesicular structures in mutant embryo are shown by asterisks. Scale bars are indicated in the image panels. Wild-type (n = 3) and mutant embryos (n = 7).

The mutant blastocysts at E3.5-E4.5 were obtained at the expected Mendelian ratio (χ^2^-test, *p* = 0.78 and 0.98 for E3.5 and E4.5, respectively) (Table 1). At the blastocyst stage, the mutant embryos were indistinguishable from the wild-type or heterozygous embryos by their gross morphology (Supplementary Fig. S2 and S3). However, at E5.5, the mutant and normal embryos showed obvious differences. The mutant embryos were found in the decidua, indicating that they could evoke decidualisation of the maternal uterine tissue; however, the embryos were smaller than the wild-type and heterozygotes. We often observed ‘empty’ decidua in which discernible embryos were absent. At E5.5, the ratio of homozygous for *Atp6v0c* became fewer than expected (*p* < 0.05) (Table 1). The wild-type embryos at E5.2 onwards, CDX2-positive extraembryonic ectoderm (ExE) and OCT3/4-positive epiblast occupy the proximal and distal regions of a cup-shaped structure (egg cylinder), respectively. They are surrounded by GATA6-positive VE. The mutant embryo differentiated ExE, epiblast, and VE at around E5.2, but ExE and epiblast apparently reduced in sizes (Fig. 1a–j). A few regions of *Atp6v0c* mutant embryos were positive for both OCT3/4 and GATA6, indicating that the patterning of epiblast and VE was impaired. These results suggest that the differentiation program for epiblast, ExE, and endoderm specification took place, however, their correct organisation was affected to some extent by the absence of the V-ATPase c-subunit function.

In the E5.2 wild-type embryos, the GATA6-positive VE layer seemed to be tightly sealed, and simple epithelium surrounded the epiblast and ExE (Fig. 1a–e). In the mutant embryos, the GATA6-positive cells did not exhibit epithelial structure but they appeared as a clump (Fig. 1f–j, white arrows, and Supplementary Fig. S3c and d). The GATA6-positive cells in the clump accumulated SOX17, another transcription factor expressed in VE^30,31^ (Supplementary Figure S3). At E4.5, cells on the surface of the inner cell mass accumulate the GATA6 and SOX17, demarcating the primitive endoderm (PrE) and epiblast. The mutant embryos showed correct expression patterns for GATA6 and SOX17 at E4.5, indicating that they could establish endoderm lineage (Supplementary Figure S3a, b). In wild-type embryos from E5.2 onwards, SOX17 distribution in VE becomes unequal. VE overlying ExE, referred to as ex(traembryonic)VE, retains SOX17, whereas the em(bryonic)VE overlying epiblast downregulates *Sox17* expression^31^. However, E5.5 mutant embryos exhibited high levels of SOX17 in the entire VE (Supplementary Figure S3c,d). This ectopic accumulation of SOX17 may reflect that reduced development of epiblast in the mutant embryos.

Electron microscopy confirmed that VE cells clustered around the distal region in mutant embryos (Fig. 1). However, unlike the wild-type VE that forms a simple epithelium (Fig. 1k–m), mutant VE cells showed no typical epithelial structures (Fig. 1n–p). In the wild-type, the apical surface of VE is rather flat and concave with microvilli, a typical apical characteristic structure. VE cells contain large vacuolar structures as known as apical vacuoles (AVs) residing at the apical side of the nuclei in wild-type embryos^27^. The AVs were prominent in wild-type VE cells (Fig. 1m, black arrows); however, a large vacuolar structure was absent in mutant VE cells, which showed only fragmented vesicles in the subapical region (Fig. 1p, asterisk).

The morphology of PE under an electron microscope showed no significant differences between the wild-type and mutant embryos (Supplementary Fig. S4). PE cells synthesize and deposit extracellular matrix proteins including laminins and collagens, thereby they contribute the assembly of Reichert’s membrane (RM)^32^. In the wild-type embryos, a large amounts of laminin α1, which is the prominent laminin in RM^33^, was highly accumulated in the abembryonic extracellular space of the VE, and lesser amounts were found between VE and ExE. We found that E5.5 mutant embryos could assemble RM positive for laminin α1, suggesting that the PE remained functional (Supplementary Fig. S5).

Electron microscopy analysis revealed that there was no increase of pyknotic cells in the KO embryos (Fig. 1n–p) suggesting that embryonic architecture was disrupted without the massive cell death.

**Figure 2:**
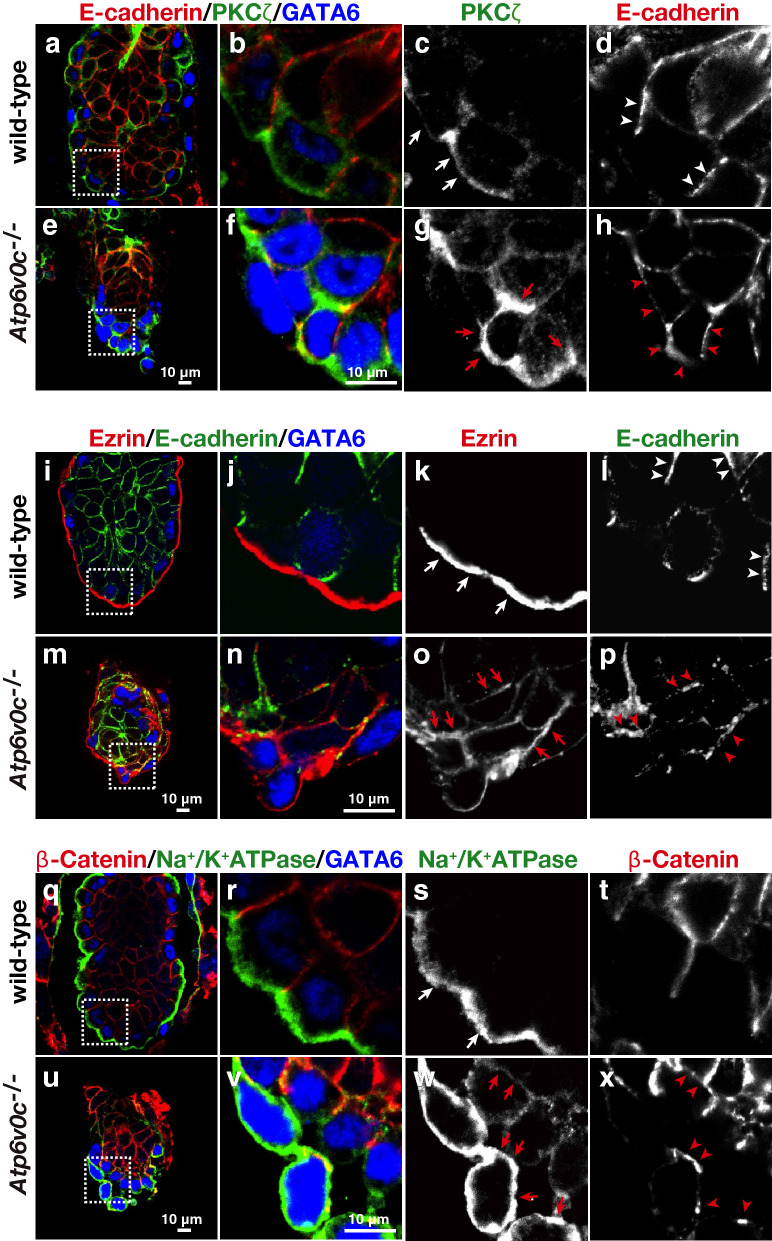
Immunofluorescence analyses of E5.5 embryos with apical and basolateral markers. The wild-type and *Atp6v0c* mutant embryos were labelled with anti-E-cadherin (red) (wild-type: n = 25, mutant: n = 6), PKCζ (green) (wild-type: n = 5, mutant: n = 4), and GATA6 (blue) antibodies (**a** to **h**). The embryos were labelled with anti-ezrin (red) (wild-type: n = 5, mutant: n = 3), E-cadherin (green), and GATA6 (blue) antibodies (**i** to **p**). The embryos were also treated with anti-β-catenin (red) (wild-type: n = 6, mutant: n = 4), Na^+^/K^+^-ATPase (green) (wild-type: n = 3, mutant: n = 4), and GATA6 (blue) antibodies (**q** to **x**). The apical markers and basolateral markers in wild-type embryos are indicated by white arrows and arrowheads, respectively. The signals of apical markers and basolateral markers in mutant embryos are indicated by red arrows and arrowheads, respectively. Scale bar, 10 µm.

The apical-basal specification in polarised cells is established by the aPKC-Par system. aPKCζ is preferentially localised in the subapical cytosol. During early embryogenesis in mice, aPKC accumulation is accompanied with the differentiation of the PrE at E4.0^34^. PKCζ was also enriched in the apical region of VE cells of wild-type E5.5 embryos (Fig. 2a–d). In *Atp6v0c*-deficient embryos, however, aPKC was more broadly distributed in the cells, suggesting the expansion and/or randomisation of the apical characteristics (Fig. 2e–h). The PKCζ images appeared curved, showing that the apical surface became rather spherical than flat (Fig. 2g). The aPKC-Par signals determine the distribution of ezrin, which connects the apical cell surface and the cortical actin cytoskeleton. Ezrin was highly restricted to the apical cell surface of wild-type embryos (Fig. 2i–l). By contrast, ezrin localised to entire plasma membrane of cells in mutant embryos (Fig. 2m–p).

E-cadherin is known to accumulate in adherens junctions, thus depicting the lateral side of cells in wild-type embryos (Fig. 2 a to d and i to l). However, E-cadherin was distributed as small dots in the presumptive basolateral cell surface of the mutant VE and frequently colocalised with PKCζ and ezrin (Fig. 2g, h, o, and p), the apical membrane residents in the VE. β-catenin, the binding partner of E-cadherin exhibited basolateral staining in wild-type embryos (Fig. 2q–t), also showed abnormal distribution in mutant embryos (Fig. 2u–x). These results suggest that the apical surface and basolateral membranes were not strictly maintained in VE cells, resulting in deformation of the epithelial architecture in the mutant embryos.

We also examined another polarity marker, Na^+^/K^+^-ATPase (Fig. 2q–x). Interestingly, we found that Na^+^/K^+^-ATPase was localised to the apical surface of the wild-type VE (Fig. 2q–t). This subcellular localisation was apparently different from that observed in most of the epithelium, including renal epithelial cells, where the sodium pump is highly restricted to the basolateral cell membrane. The opposite distribution along the apical-basal axis was also described in the retinal pigmented epithelium and choroid plexus (for review, see^35^). In *Atp6v0c*-mutant embryos, however, Na^+^/K^+^-ATPase accumulated in entire cell surface of the cells located in the outer side of the embryos. In the inner VE cells, lesser amounts of Na^+^/K^+^-ATPase were found, but their cellular distribution indicate loss of apical-basal polarity (Fig. 2u–x). Taken together, these observations suggest that *Atp6v0c* deficiency results in the loss of the apical-basolateral organisation in the VE.

### The luminal acidification of endocytic compartments was defective in *Atp6v0*c null embryos

**Figure 3:**
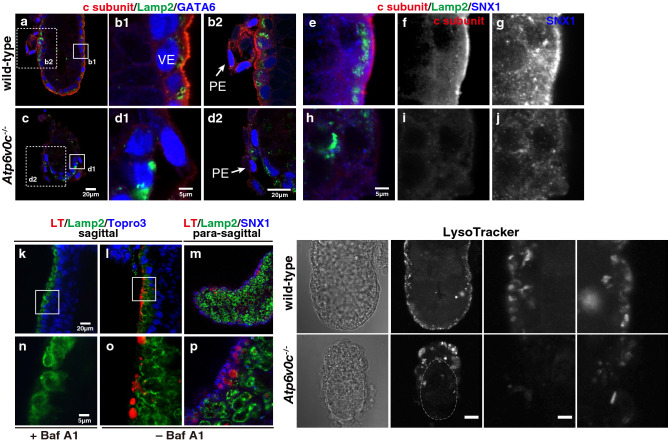
Acidification of intracellular compartments of E5.5 embryos required the c-subunit of V-ATPase. The signal of the c-subunit (red) (wild-type: n = 9, mutant: n = 12) accumulated at the apical region of VE cells in E5.5 embryos (**a** to **d**). Lamp2 (green) and GATA6 (blue) signals are also shown. The boxed areas of b1 and d1 are enlarged images showing the signals of c-subunit in VE cells, and the boxed region of b2 and d2 are those of PE cells (shown by the arrow). The c-subunit (red) signal overlapped with the early endosome marker, sorting nexin1 (SNX1) in E5.5 wild-type embryos (n = 3) (**e** to **g**). The localisation of SNX1 in mutant embryos appeared dispersed (n = 4) (**h** to **j**). VE, visceral endoderm; PE, parietal endoderm. The E6.0 wild-type embryos were labelled with LysoTracker (LT) in the presence (**k** and **n**) (n = 4) or absence of bafilomycin A1 (Baf A1) (n = 14) (**l**, **m**, **o**, and **p**). The sagittal images (LT in red, Lamp2 in green, and Topro3 in blue) and para-sagittal images (LT in red, Lamp2 in green, and SNX1 in blue) are shown. The signal of LysoTracker was surrounded by the signal of SNX1 (**m** and **p**). LysoTracker staining of E5.5 wild-type (n = 17) (**q** and **r**) and *Atp6v0c* mutant embryos (n = 3) (**s** and **t**). The boxed regions in r and t are enlarged, and scale bars in panels a, c, b2, d2, k–m, and q–t, are 20 µm, scale bars in panels b1, d1, e–j, n–p, and 1–4 are 5 µm.

We examined the distribution of the V-ATPase c-subunit by immunostaining of whole embryos. At E4.5, PrE exhibited accumulation of c-subunit, whereas OCT3/4-positive-inner cell mass (ICM) did less amount of this V-ATPase component. The signal of c-subunit was also positive in both mural TE positioned away from the ICM and polar TE cells overlying the blastocoel (Supplementary Fig. S6). In E5.5 embryos, the c-subunit was more abundantly accumulated in VE cells than in the ExE and epiblast. In VE cells, as well as PrE, of the wild-type embryos, the subapical region was highly positive for the c-subunit (Fig. 3a and b1). The signals of c-subunit in PE cells were also observe in wild-type embryos (Fig. 3b2). By contrast, *Atp6v0c*-mutant embryos showed only background staining for the c-subunit in either VE or PE (Fig. 3c and d1-2).

The AVs, which are rich in the lysosome-marker protein Lamp2, accumulate less c-subunit (Fig. 3a,b1,e,f). SNX1, the early endosome marker, co-localised with the c-subunit, indicating that the c-subunits were mainly associated with the early endocytic compartments in the subapical cytoplasm (Fig. 3e–g). By contrast, the mutant VE accumulated reduced amounts of SNX1 and Lamp2 (Fig. 3h–j).

We then examined the luminal acidification of intracellular organelles of wild-type embryos at around E5.5 to E6.0 using LysoTracker (LT) probes, which selectively accumulate in cellular compartments with acidic pH. The early endocytic compartments in the subapical cytosol were labelled with LT, which disappeared when the embryos were incubated with bafilomycin A1 (BafA1), a specific and potent inhibitor of V-ATPase. These results suggest that the luminal acidic pH of intracellular compartments is generated by V-ATPase in the E5.5 embryo (Fig. 3k–p,q,r, boxed regions 1 and 2). The fluorescence intensity of LT in mutant embryos was faint; the signals observed were probably those from the maternal tissue isolated together with the embryo (Fig. 3s,t, boxed regions 3 and 4).

### Endocytic uptake was defective in *Atp6v0c* null embryos

**Figure 4:**
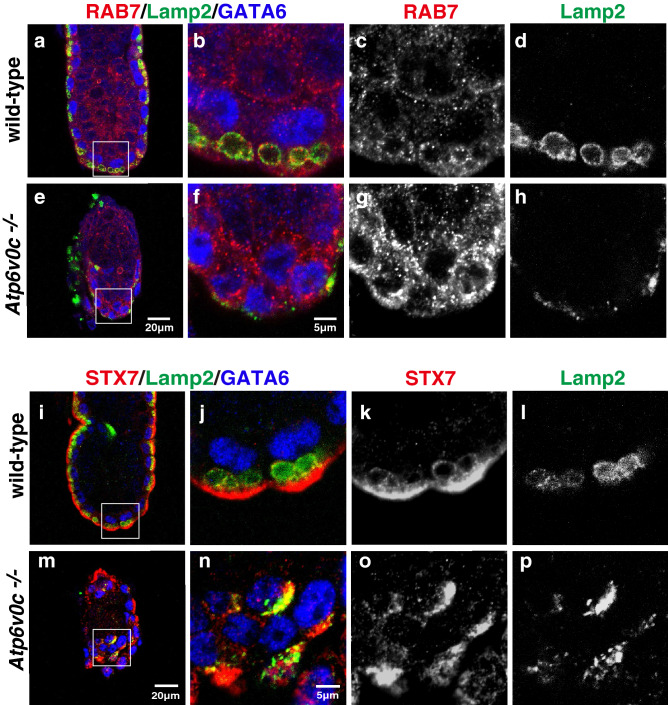
The morphologies of late endosomes/lysosomes in VE cells of E5.5 embryos. Localisation of endocytic organelle markers, RAB7 (red) and Lamp2 (green) in wild-type (**a–d**) (n = 3) and *Atp6v0c* mutant embryos (**e**–**h**) (n = 7). Localisation of syntaxin7 (Stx7, red) and Lamp2 (green) in wild-type (**i–l**) (n = 11) and *Atp6v0c* mutant embryos (**m–p**) (n = 3). VE cells were labelled with GATA6 (blue). Large apical vacuoles labelled by Lamp2 were well developed in the wild-type VE cells, whereas Lamp2 signal was dispersed and the spherical structures were not observed in mutant embryo. Syntaxin7 co-localised with Lamp2 and was also enriched in the apical region of wild-type VE cells. The apical localisation pattern of syntaxin7 was also disturbed in mutant GATA6-positive VE cells. Scale bars are indicated in each panel.

The VE is a polarised-absorbing epithelium that actively internalises various molecules, including transferrin, immunoglobulins, lipoproteins, and albumin^36^. We first examined the morphology of organelles in the endocytic pathway in VE cells. Lamp2, a membrane protein localised mainly to the lysosome, was detected on the AV membrane (Fig. 4a,b,d; i, j, and l). The late endosome/lysosome SNARE, syntaxin7, was localised to the ring-like Lamp2-positive AV membranes, and abundantly accumulated at more apical regions (Fig. 4i,j,k). RAB7, a small GTP-binding protein required for microautophagy in VE cells^3^, appeared as dots in both the apical and basolateral cytoplasm; RAB7 signals often decorated the AV membranes (Fig. 4a,b,c). However, in mutant embryos, the signals of Lamp2 decreased and exhibited a dispersed pattern (Fig. 4e,f,h; m, n, and p). The apical concentration of syntaxin7 signals was less prominent (Fig. 4m,n,o). These observations were consistent with the morphology of intracellular organelles of VE cells under electron microscopy (Fig. 1m,p), indicating that the integrity of the AV structure was disrupted. RAB7 signal was elevated in mutant VE cells (Fig. 4e,f,g), suggesting certain trafficking blocks in the endocytic pathway which lead to abnormal accumulation of RAB7-positive vesicles in VE cells.

**Figure 5:**
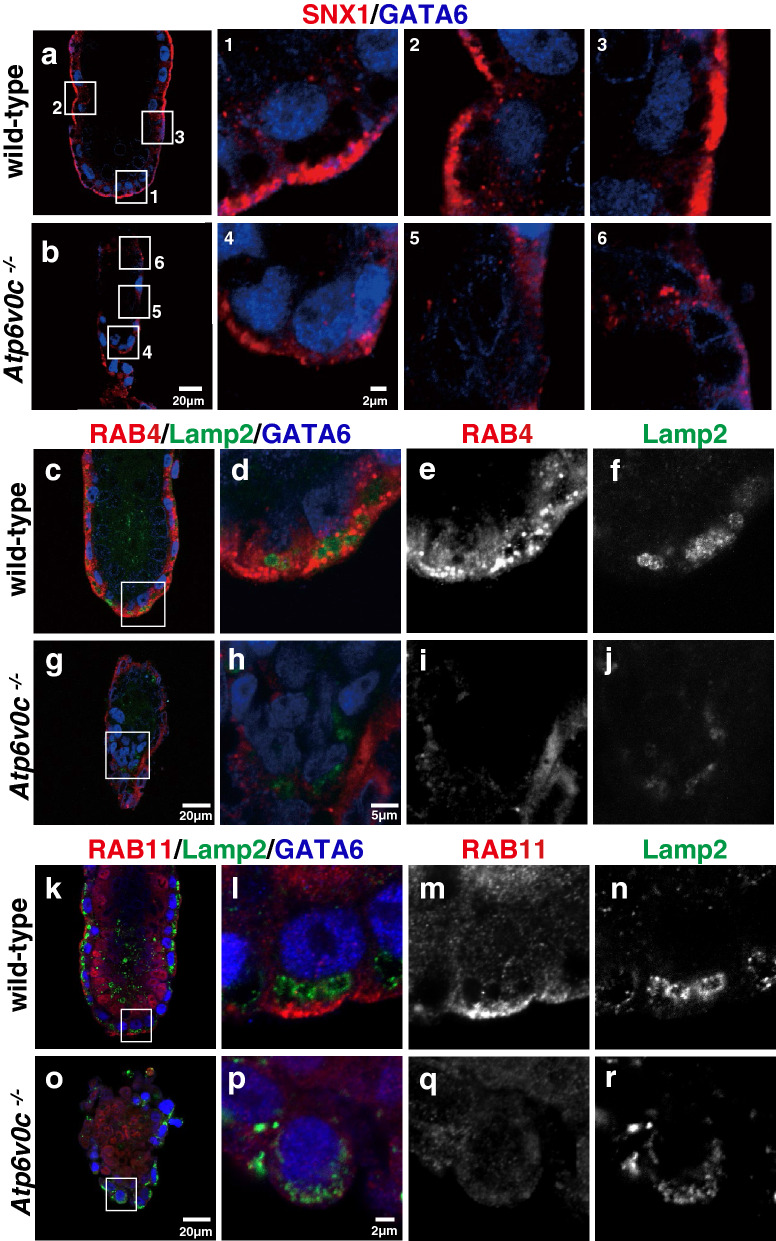
Early endosome/lysosome in VE cells of E5.5 embryos. Localisation of the early endosome marker, SNX1 (red), in wild-type (**a**, and boxed regions 1 to 3) (n = 3) and *Atp6v0c* mutant embryos (**b**, and boxed regions 4 to 6) (n = 4). VE cells were labelled with GATA6 (blue). Localisation of the recycling endosome marker, RAB4 (red), and Lamp2 (green) in wild-type (**c–f**) (n = 9) and *Atp6v0c* mutant embryos (g–j) (n = 4). Localisation of the recycling endosome marker, RAB11 (red), and Lamp2 (green) in wild-type (**k–n**) (n = 18) and *Atp6v0c* mutant embryos (**o–r**) (n = 13). Scale bars are indicated in each panel.

The distribution of early endosomes in mutant VE cells was also affected. Sorting nexin 1 (SNX1)^37^ was detected in both apical and basolateral regions but was enriched in the apical region of wild-type VE cells (Fig. 5a, and boxed regions 1–3). However, SNX1 expression decreased in mutant cells (Fig. 5b, and boxed regions 4–6). RAB4, which is required for directing early endosomes to the recycling endosome compartment, also diminished and exhibited a smear distribution pattern in mutant VE cells (Fig. 5g–j) instead of the dot-like pattern observed in wild-type cells (Fig. 5c–f). The recycling endosome marker RAB11 was localised at the apical region in wild-type embryos (Fig. 5k–n), but the signal was below the detection level in *Atp6v0c* mutant embryos (Fig. 5o–r).

**Figure 6:**
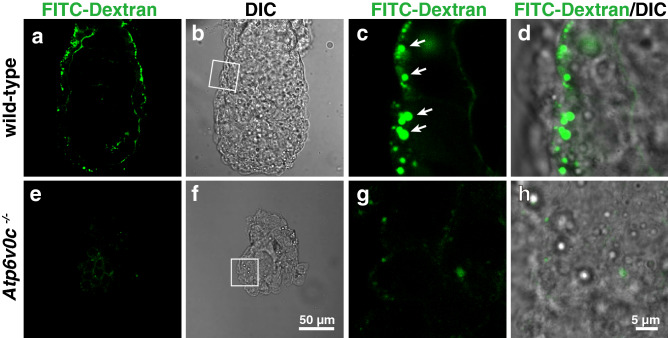
Endocytosis of FITC dextran in VE cells. E5.5 embryos were in utero labelled with fluorescent dextran. The embryos were fixed and observed under a confocal microscope. Wild-type and heterozygous embryos could internalise fluorescent dextran (arrows) via endocytosis (**a** to **d**, arrows) (n = 15), whereas the fluorescent signals in *Atp6v0c* mutant embryos (**e** to **h**) (n = 3) were under the detection level. Scale bars are indicated in each panel.

As the morphology of the organelles of the endocytic pathway was significantly affected, we examined endocytic activity. The wild-type mouse embryo took up fluorescein (FITC)-dextran from the maternal circulation when the marker dye was intravenously introduced into pregnant mothers (in utero labelling). The internalised FITC-dextran signals were observed in VE cells of wild-type embryos 30 min after intravenous administration (Fig. 6a–d). However, the fluorescent signal in mutant embryos was significantly low (Fig. 6e–h), indicating that the endocytic activity was defective in *Atp6v0c*-deficient embryos. This result was consistent with our previous observation that the outgrowth of *Atp6v0c*-deficient blastocyst failed to internalise FITC-dextran^16^.

## Discussion

The loss of the c-subunit of V-ATPase results in severe defects in the VE of early embryos. The VE exhibits clear polarity; its apical surface faces the maternal circulation and the basal side faces the embryo proper. This highly polarised epithelial tissue has important nutritional roles, and influences the development of other embryonic tissues^38^. The specialised repertory of VE, known as the anterior visceral endoderm, is an organising centre for the anterior–posterior specification at E5.2–E6.5 embryos^39,40^. *Atp6v0c* mutant embryos suffer severe dysfunction in VE and were unable to develop beyond the egg cylinder stage.

In our previous study, we showed that he mutant embryos lacking the V-ATPase proteolipid subunit could still develop up to the E3.5 blastocyst stage and contained bafilomycin A1-sensitive acidic compartments similar to the wild-type^16^. We detected *Atp6v0c* mutant embryos at E4.5 at the Mendelian ratio in this study. It is possible that a maternal pool of the proteolipid and/or its transcripts are able to support the preimplantation growth.

How V-ATPase is involved in cell polarity establishment and/or maintenance is yet questionable. One possibility is that the luminal acidification of endocytic compartments is essential for polarised vesicle trafficking. Various studies in flies, nematodes, and cultures of mammalian epithelial cells suggest that endocytic trafficking plays an important role in establishing cell polarity^7^. Transcytosis, a highly regulated coupling of endocytosis, sorting, and exocytosis, is a prerequisite for establishing and maintaining the apical-basal polarity^41^. Endocytosed molecules pass through a series of endosomal compartments where some molecules are sorted and sent back to the basolateral surface while others are transported to the apical cell surface (for review, see^42^).

Luminal acidification and endocytic activity were markedly decreased upon loss of this proton pump. The severe defect in endocytosis readily compromises membrane trafficking, thus disrupting the apical-basal polarity of the epithelial tissue. The VE of the peri-gastrulation stage is highly active in endocytosis from the apical cell surface. The robust activity of apical endocytosis in VE cells requires V-ATPase function. In *Atp6v0c* mutant embryos, the apical markers of VE cells expanded and the basolateral characteristics were severely compromised. Active endocytosis, a high flow of de novo synthetic pathway, and recycling flow from the early endosome are tightly coordinated to correctly maintain the proportion of membranes. Defective endocytosis from the apical cell surface upon inactivation of V-ATPase results in expansion of the apical membrane.

Dysregulation in the early phases of the endocytic pathway leads to severe defects in the VE. Disabled-2 (Dab2) is an adapter molecule that associates with various signalling receptors. Its function is important for endocytosis of a class of cell surface molecules, including low density lipoprotein receptor-related proteins. The ICM lacking *dab2* can differentiate the epiblast and primitive endoderm, showing that its deficiency does not affect the normal specification and cell fate determination of ectodermal and endodermal lineages in the ICM. However, in *dab2* mutant embryos, GATA6-positive primitive endoderm cells remain intermingled with epiblast cells expressing OCT3/4. Thus, Dab2 is necessary for positioning control of primitive endoderm cells^43^. GATA6-positive cells of *Atp6v0c* mutant embryos were separated from the OCT3/4-positive epiblast, indicating that the endoderm specification was executed in *Atp6v0c* mutant embryos unlike the mutants lacking *Dab2*.

*Atp6v0c* mutant embryos failed to exclude the expression of *Sox17* in the emVE. Downregulation of exVE-specific marker molecules is dependent on the Nodal^44^. VE secretes converting enzymes which are the prerequisite for activation of NODAL protein. Loss of V-ATPase function affects the integrity of endocytic as well as exocytic pathways in VE and epiblast, thereby it leads failure in secretion of the convertases as well as NODAL itself. Multiple signalling pathways including NODAL and other secreted molecules are playing essential regulatory roles in patterning and growth of the embryo throughout the egg cylinder stages. Dysregulation of the Nodal signalling and others may be an underlying mechanism for the developmental defects associated to the loss of *Atp6v0c* function*.*

Among the various endocytic proteins, RAB4 expression significantly reduced in VE cells of *Atp6v0c* mutant embryos. RAB4 showed dot-like patterns in the wild-type embryos but appeared smear-like in mutant embryos, most likely reflecting its cytosolic distribution. Hurtado-Lorenzo et al. previously demonstrated using renal epithelial cells that recruitment of the small GTPases Arf6 and ARNO from the cytosol to endosomal membranes was driven by the V-ATPase-dependent intra-endosomal acidification^21^. Arf6 interacts with the c-subunit and ARNO with the a2 isoform, consistent with the V_0_ membrane sector. It is possible that the recruitment of RAB4 to the membranes of apical endocytic compartments requires luminal acidic pH or the subunits of the V_0_ membrane sector of V-ATPase.

V-ATPase may also be involved in the regulation of apical endocytosis. Sabatini and colleagues demonstrated that lysosomal V-ATPase plays an important role in the mechanistic target of rapamycin (mTOR) signalling^45^. In *Drosophila*, the control of apical protein uptake depends on V-ATPase/mTOR signalling. RNA interference-mediated silencing of V-ATPase function results in the failure of apical endocytosis of wing imaginal disk epithelial cells, ultimately causes tissue disorganisation^46^.

Another intriguing possibility of the mechanism underlying the polarity control of VE is that V-ATPase may interact with the cell polarisation machinery such as aPKC-Par proteins or their downstream components Rac and Rho small GTPases. Genetic ablation of the Vo accessory protein ap2/(pro)renin receptor in retinal pigmented epithelium (RPE) results in its delamination, owing to defects in the apical-basal cell polarity in the epithelium. ap2 and Par3, key regulators of cell polarity, interact with each other, showing a mechanistic link between Par-aPKC signalling and V-ATPase function^26^. RPE cells share several common characteristics with VE cells, including the apical localisation of Na^+^ pump^35^. We were unable to establish colocalisation of Par3 and V-ATPase in the VE of the embryonic tissue (data not shown). Thus, it remains to be clarified whether the physical interaction of Par components and the V-ATPase complex is a general or restricted repertoire of specific epithelium.

## Materials and Methods

### Animals

All animal procedures were approved by the Committees of ISIR, Osaka University and Doshisha Women's College, and performed in accordance with the institutional and national guidelines. In addition, all the animal studies were in compliance with ARRIVE guidelines. C57Bl/6 and ICR mice were purchased from SLC Japan. The animals were provided with food and water ad libitum.

The mouse *Atp6v0c* locus was modified by homologous recombination in mouse embryonic stem cells. A targeting vector was constructed using a mini-transposon carrying *FRT*, *lox*, and *neo* elements and a bacterial artificial chromosome (BAC) containing the *Atp6v0c* locus^47^. The targeting construct was introduced into mouse embryonic stem cells R1^48^, and homologous recombination was identified by polymerase chain reaction (PCR). The chimeric animals were generated by injecting homologous recombinants into the blastocoels of C57Bl/6 embryos. *Atp6v0c*^targeted^ mice were crossed with FVB/N-Tg (EIIa-cre) C5379Lmgd/J strain expressing the Cre recombinase during early development to remove exons 2 and 3 and to generate *Atp6v0c*^+/–^ animals. *Atp6v0c*^+/–^ mice were further crossed with wild-type C57Bl/6 mice to remove the Cre transgene, and a cohort of *Atp6v0c*^+/–^ mice was established (Supplementary Fig. S1).

### Genotyping

The embryos were lysed in 10 µL of Quick Extract (EPICENTRE) and used as a template for PCR. The primer set ATP6c_wt_Fw (5′-AGGCTCTCCTGGCCCAGCCGCCTCTCC-3′) and EX2RV(R03) (5′-TGGATTCATGATCAGCTCTGGC-3′) were used to detect the wild-type allele, which yielded a 381 bp product. The primer set GH133 (5′-GAGTCTCGATCGAGGTCGACAT-3′) and TA-Rv1 (5′-CAGAAGGAGGTGACACCGTGGGA-3′) were used to detect the knockout allele, which gave an 832 bp product.

### Immunofluorescence labelling of the embryos

Embryos were obtained from pregnant females anaesthetised with isoflurane and subjected to fixative perfusion (4% paraformaldehyde [PFA] in phosphate-buffered saline [PBS]). For immunohistochemistry, the fixed embryos were incubated with primary and secondary antibodies in a blocking solution containing 0.05% Tween-20, 0.5% TSA blocking reagent (PerkinElmer), and 1% normal donkey serum in PBS. Rabbit anti-SNX1, anti-RAB7, and anti-syntaxin7 antibodies^37,49^ were used at 2.4, 15, and 4.1 µg/mL, respectively, in the blocking solution. Rat anti-Lamp2 monoclonal antibodies were obtained from Developmental Study Hybridoma Bank and used at 4.4 µg/mL. Anti-GATA6 (R&D, 1:50), anti-RAB4 (Upstate, 1:100), anti-ezrin (Cell Signalling Technology, 1:100), anti-E-cadherin (Takara, 1:500), anti-Na^+^/K^+^-ATPase (Rockland, 1:100), anti-PKCζ (Santa Cruz, 1:100), anti-β-catenin (Cell Signalling Technology, 1:100), anti-CDX2 (Cell Signalling Technology, 1:50), anti-OCT3/4 (R&D, 1:50), anti-SOX17 (R&D, 1:500), and anti-laminin α1 (R&D, 1:50) were diluted in the blocking solution and used as primary antibodies. Fluorescein-, Cy3-, and Cy5-conjugated secondary antibodies were obtained from Jackson ImmunoResearch, reconstituted in 40% glycerol according to the manufacturer’s recommendations, and used at 1:100, 1:500, and 1:250 dilutions, respectively. Embryos were staged according to the dissection time (noon of the vaginal plug as E0.5) and morphology.

### In utero endocytic labelling

At E5.5, pregnant females were administered 100 µL of 25 mg/mL fluorescent dextran (M. W. 70,000, aldehyde fixable) from the tail vein, as previously described^3^. After 30 min, mice were anaesthetised with isoflurane. Embryos were obtained, fixed with 4% PFA, and then processed for immunofluorescence microscopy after washing several times in PBS. After image recording, the embryos were genotyped as described above.

### Embryo culture and LT staining

Deciduae were excised from E6.2 pregnant females sacrificed by cervical dislocation, and placed in Dulbecco’s modified Eagle’s medium (DMEM) containing 10% foetal bovine serum (FBS) and 25 mM HEPES (pH 7.5). Embryos were dissected in the same medium under a stereomicroscope and cultured in a 1:1 mixture of DMEM and rat serum under 5% CO_2_ and 95% air at 37 °C for 30 min^50^. The embryos were then incubated in a medium containing 1 µM LT (Molecular Probes, fixable) for 30 min. The same experiment was performed in the presence of bafilomycin A1 (WAKO) at 100 nM or 1 µM concentration. After LT labelling, the embryos were fixed with 4% PFA, and processed for immunostaining or observation under a microscope.

### Microscopy

Immunostained or fluorescent probe-labelled embryos were immersed in a 1:1 mixture of 40% glycerol and VECTASHIELD (Vector Laboratories). Whole embryos were mounted in 0.1% gellan gum (Sigma-Aldrich) in PBS plus 40% glycerol in glass-bottom 35-mm dishes. Immunofluorescence samples were viewed under a confocal laser scanning microscope (Zeiss LSM 510 or 800).

For electron microscopy, E5.5 decidua were fixed in 2.5% glutaraldehyde and 4% PFA in 0.1 M potassium phosphate buffer (pH 7.4), and electron microscopy was performed at the TOKAI electron microscopy analysis centre.

All images were processed using Adobe Photoshop for presentation. Contrast/brightness was adjusted on the entire images, and the processing conformed to all the guidelines specified in the Image integrity and standards of the Journal. The original data are available upon reasonable request.

## Supplementary Information

Supplementary Information 1.

Supplementary Information 2.
