# Supplementary material for: Vacuolar-type proton ATPase is required for maintenance of apicobasal polarity of embryonic visceral endoderm
Source: Sci Rep. 2021 Sep 29;11:19355. doi: 10.1038/s41598-021-98952-3 (PMC8481250; doi:10.1038/s41598-021-98952-3)
Supplement: Supplementary file 1 — Supplementary Information 1. [file 41598_2021_98952_MOESM1_ESM.pdf]

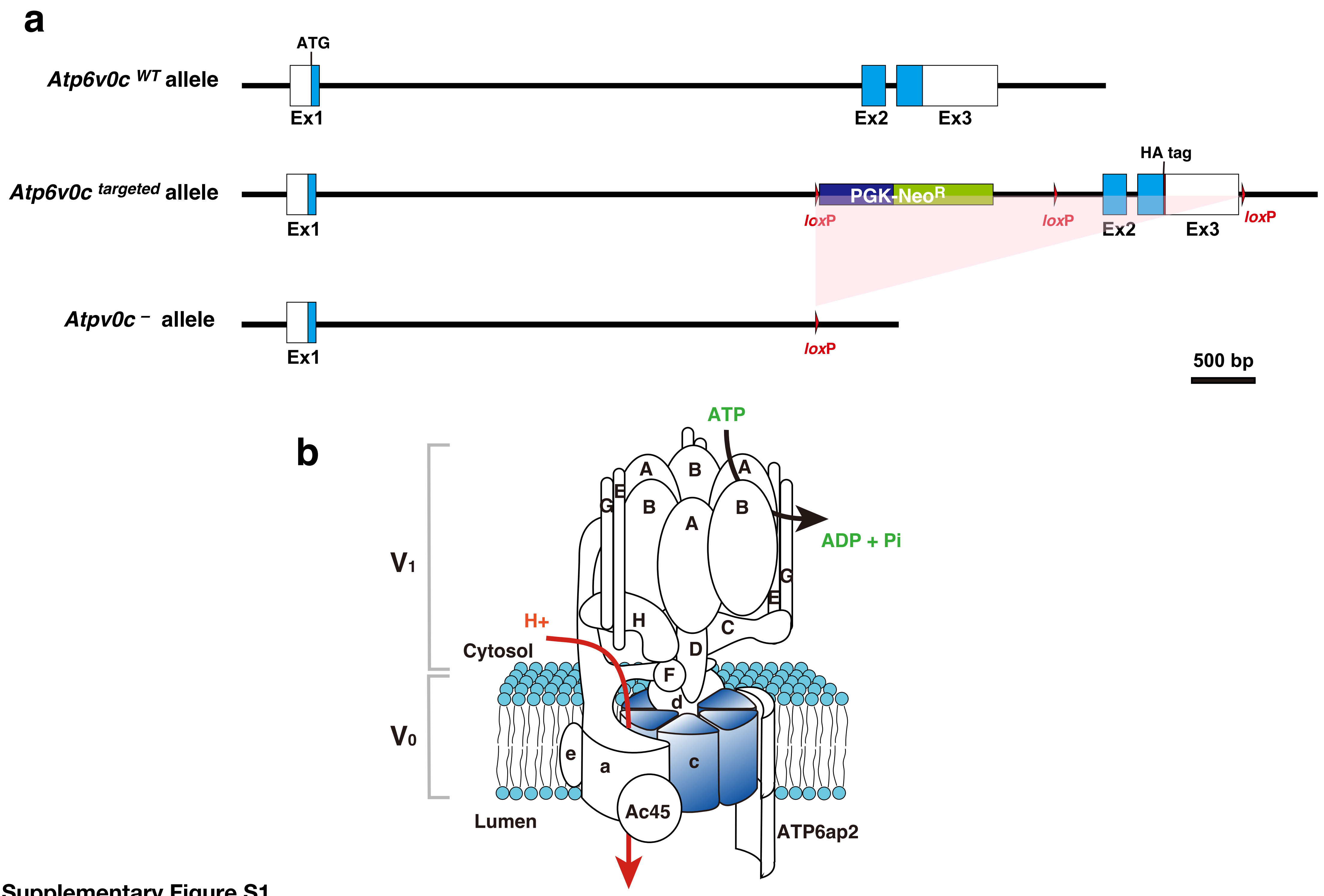

Supplementary Figure S1

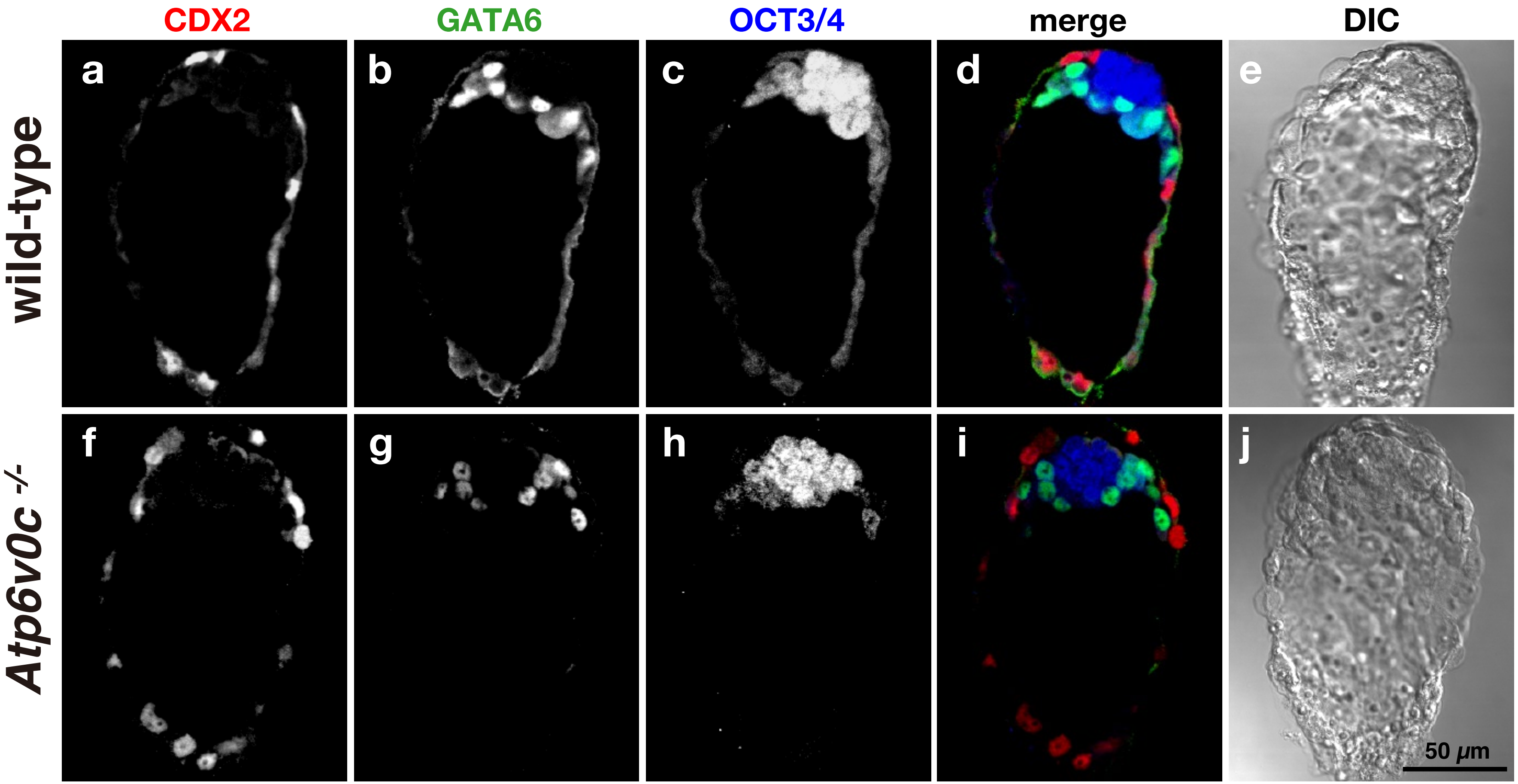

Supplementary Figure S2

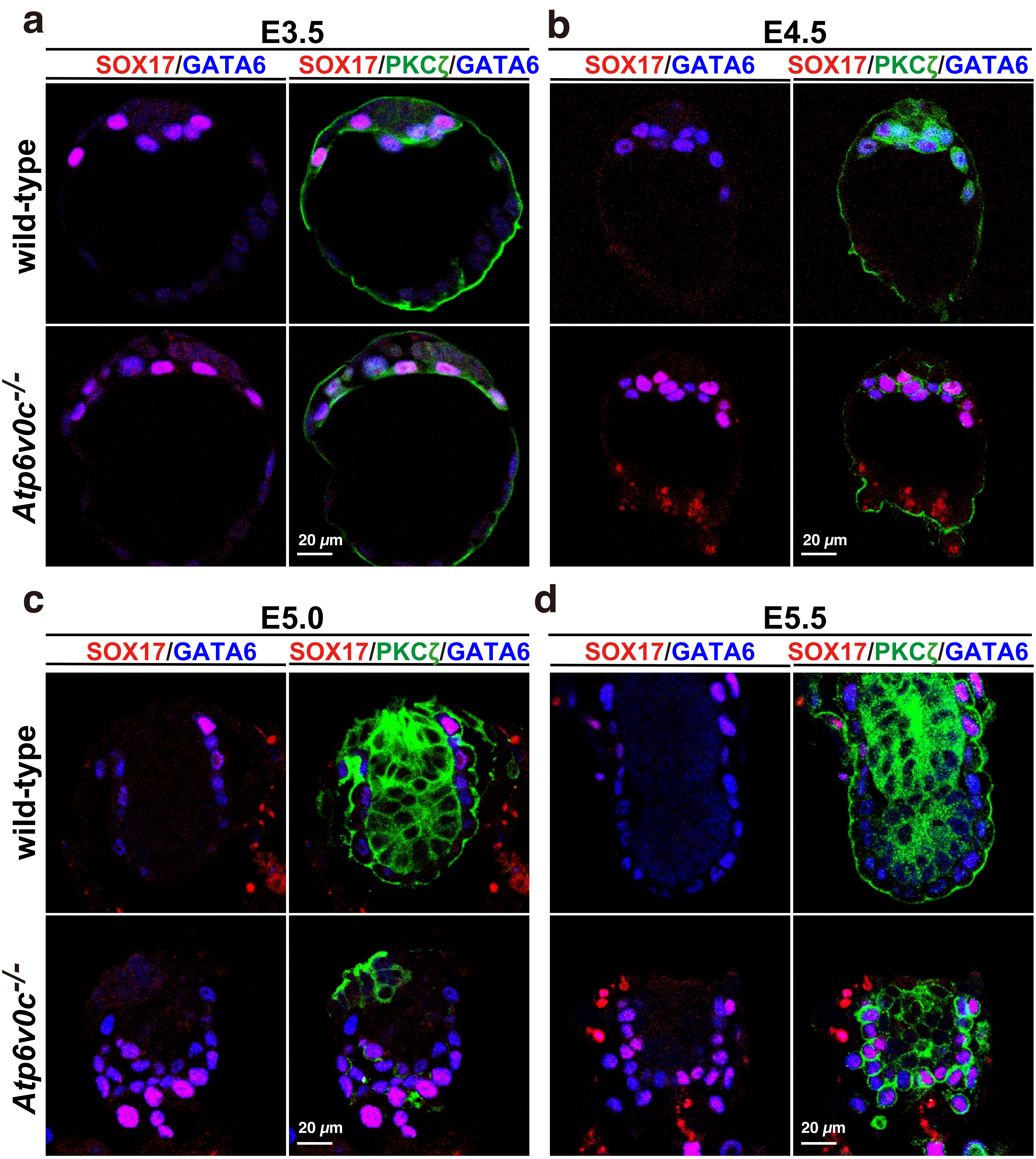

Supplementary Figure S3

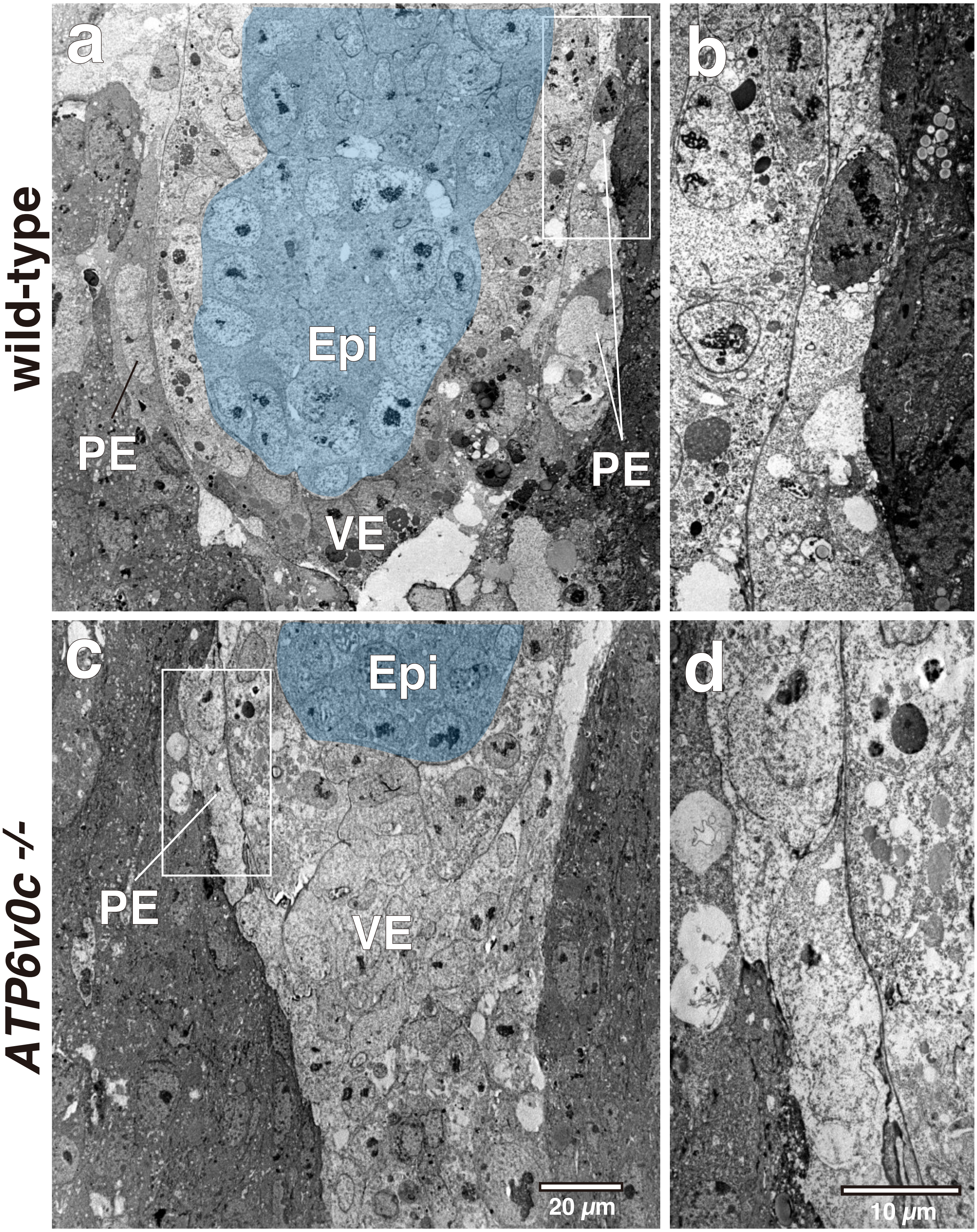

Supplementary Figure S4

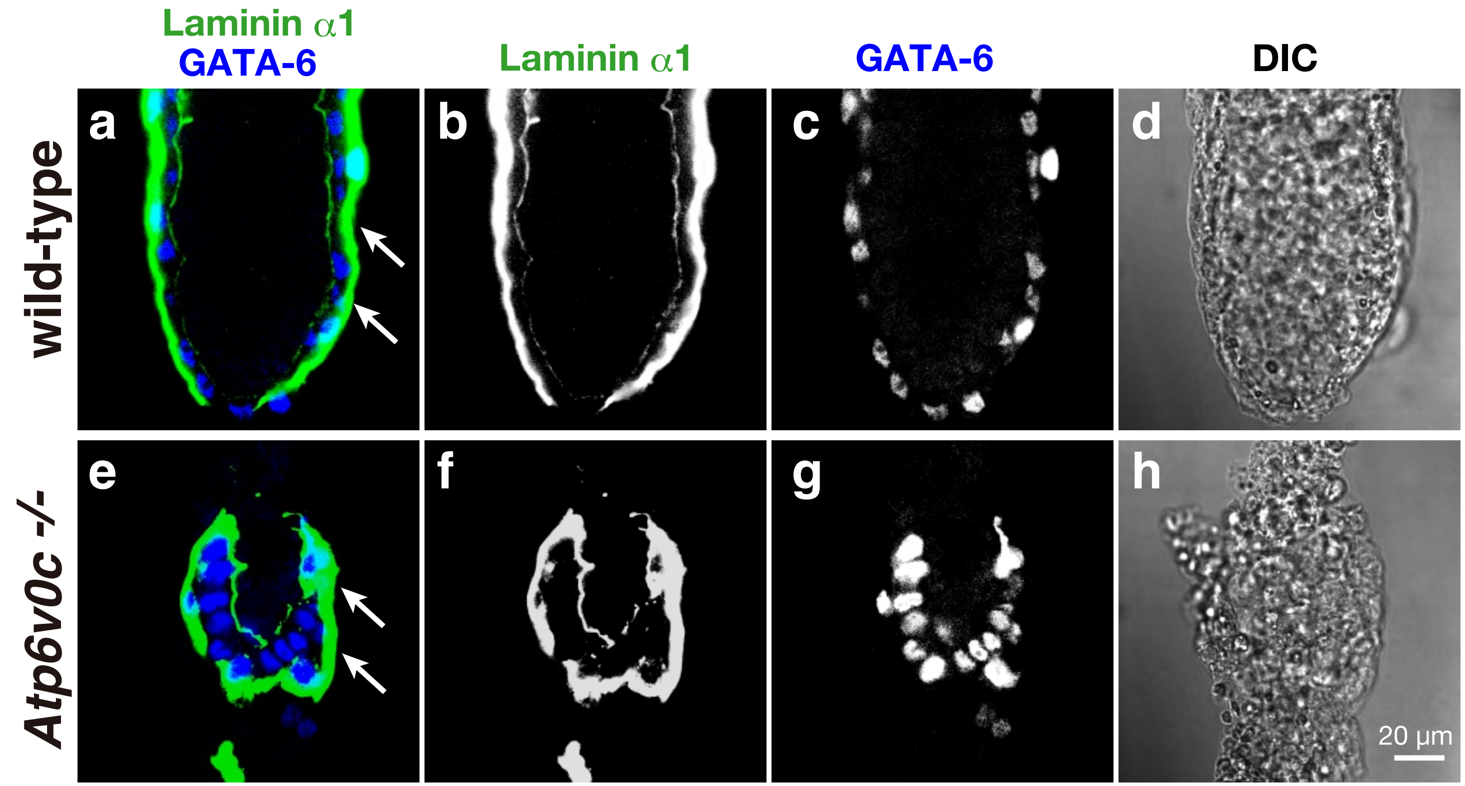

Supplementary Figure S5

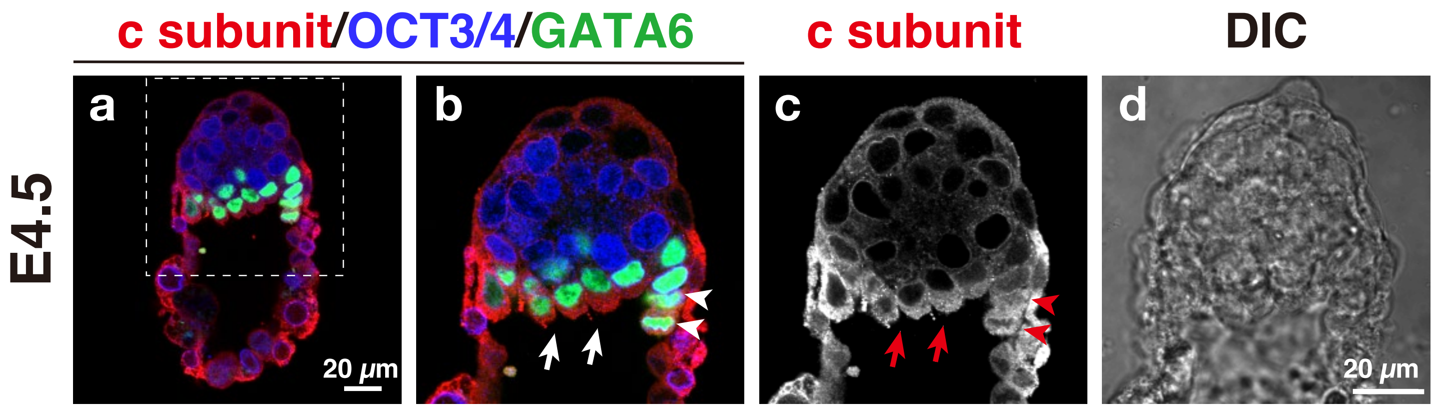

Supplementary Figure S6
