# Supplementary material for: Vacuolar-type proton ATPase is required for maintenance of apicobasal polarity of embryonic visceral endoderm
Source: Sci Rep. 2021 Sep 29;11:19355. doi: 10.1038/s41598-021-98952-3 (PMC8481250; doi:10.1038/s41598-021-98952-3)
Supplement: Supplementary file 2 — Supplementary Information 2. [file 41598_2021_98952_MOESM2_ESM.pdf]

Supplementary Figure S1. Genetic modification of the mouse *Atp6v0c* locus.

The wild-type allele of *Atp6v0c* is shown at the top, and the open-reading frame of the c-subunit is shown in blue. The translation initiation site (ATG) is also shown. A gene-targeting construct, wherein an *FRT-lox-neo* cassette was placed in intron 1-2 and the third loxP element in the 3′-UTR region was introduced into the *Atp6v0c* locus of the embryonic stem cell line R1. Chimeric animals were generated by injecting the homologous recombinants into the blastocoels of C57Bl/6 embryos. *Atp6v0c*^{targeted} mice were crossed with FVB/N-Tg (EIIa-cre) C5379Lmgd/J strain expressing the Cre recombinase during early development to remove exons 2 and 3 and to generate *Atp6v0c*$^{+/-}$ animals (a). The V-ATPase complex is composed of a peripheral domain (V_1), which is responsible for ATP hydrolysis, and an integral domain (V_0), which is involved in proton translocation across the membrane. The proteolipid c-subunit encoded by *Atp6v0c* is shown in blue. Schematic model of V-ATPase was drawn by Adobe Illustrator ver. 19 (CC2015) (b).

Supplementary Figure S2. Cell fate specification in *Atp6v0c* mutant embryos.

CDX2, GATA6, and OCT3/4 expression in E4.5 embryos was examined by immunofluorescence analysis. Differential interference contrast (DIC) images are shown. The mutant embryos (n = 12) could differentiate the GATA6-positive primitive endoderm and the OCT3/4-positive epiblast as wild-type embryos (n =13). Scale bar, 50 µm.

Supplementary Figure S3. Endoderm differentiation in *Atp6v0c* mutant embryos.

At E3.5 (a), E4.5 (b), E5.0 (c), and E5.5 (d), the wild-type and *Atp6v0c* mutant embryos were labelled with anti-SOX17 (red), PKCζ (green), and GATA6 (blue) antibodies. The mutant blastocysts at E3.5-E4.5 were indistinguishable from the wild-type embryos by their gross morphology (a and b). In *Atp6v0c*$^{-/-}$ mutant embryos at E5.0 to E5.5, GATA6- and SOX17-positive VE cells exhibited defects in apical-basolateral polarity. Embryo number observed at E3.5: wild-type, n = 1; heterozygous, n = 10; mutant, n = 7; E4.5: wild-type, n = 3; mutant, n = 4; E5.0: wild-type, n = 8; mutant, n = 6; E5.5: wild-type, n = 7; mutant, n = 3. Scale bars, 20 µm.

Supplementary Figure S4. Transmission electron microscopy of E5.5 PE cells.
The decidua at E5.5 of the wild-type (a and b) (n = 3) and *Atp6v0c* mutant embryo (c and d) (n = 7) were examined under transmission electron microscopy. The boxed regions of parietal endoderm (PE) in panel a and c are enlarged (b and d). PE, parietal endoderm; VE, visceral endoderm; Epi, epiblast. Scale bars for panels a and c are 20 μm and panels b and d are 10 μm.

Supplementary Figure S5. Expression of laminin α1 in E5.5 embryos
Wild-type (a - d) (n = 10) and mutant embryos (e - h) (n = 6) E5.5 embryos were isolated and labelled with antibodies against Laminin α1 (green) and GATA6 (blue). The arrows indicate the Reichert's Membrane. Scale bars, 20 μm.

Supplementary Figure S6. Localization of c-subunit in E4.5 late blastocyst.
The wild-type blastocysts at E4.5 (n = 8) were isolated and labelled with antibodies against c-subunit (red), GATA6 (green) and OCT3/4 (blue) (a - d). The signals of c-subunit appeared to be enriched in the apical region of primitive endoderm including visceral endoderm (arrows) and parietal endoderm (arrowheads) at E4.5 (b and d). Images are representative of x and y blastocysts at E4.5. Scale bars, 20 μm.
